# Predictors of Treatment Completion Among Clients Involved with the U.S. Public Mental Health System

**DOI:** 10.1192/j.eurpsy.2025.487

**Published:** 2025-08-26

**Authors:** S. Yampolskaya, L. Callejas, C. Walker-Egea

**Affiliations:** 1 Child & Family Studies, University of South Florida , Tampa, United States

## Abstract

**Introduction:**

Research indicates that for psychiatric services to be generally effective, individuals have to complete the treatment. Although treatment completion does not necessarily equate to treatment success, it has been found to consistently lead to improved outcomes including improved rates of recovery and reduced risk of involvement with the justice system and other adverse outcomes. Treatment non-completion, on the other hand, has been associated with a higher risk of readmissions for inpatient care, legal, and other health-related problems (*Dreyer et al.* S Afr J Psychiatr 2020; 26 a1255). These findings underscore the importance of examining factors associated with treatment completion.

**Objectives:**

The goal of the study was to identify predictors for treatment completion among individuals receiving psychiatric services from state-administered behavioral health systems in Indiana, USA. Three domains of predictors were examined, including sociodemographic characteristics, comorbid conditions, and the type of treatment patients received.

**Methods:**

Statewide records from merged administrative data sets were used for this analysis. Multivariate logistic regression was conducted using a sample of 15,412 adults receiving behavioral health services in fiscal year 2020.

**Results:**

Among demographic characteristics only race was associated with treatment completion. Individuals who were non-Hispanic White were 20% more likely to complete their treatment compared to their minority (e.g., Black, Hispanic) counterparts (*p* < .05, OR = 1.20). Having more than one mental health disorder was associated with 16% decreased odds of completing treatment (*p* < .05, OR = 0.87). Individuals who were institutionalized for psychiatric conditions had 26% decreased odds of completing the treatment (*p* < .05, OR = 0.80), but those who received inpatient substance abuse related care were over four times more likely to complete their treatment (*p* < .05, OR = 4.61). Finally, individuals who attended any self-help groups had 63% greater odds of completing the treatment.

**Conclusions:**

Findings suggest that individuals who simultaneously received substance abuse and mental health treatment had the greatest chances to complete treatment successfully. Study implications also suggest that self-help or support groups should be promoted as ways to motivate patients to adhere to treatment.

**Disclosure of Interest:**

None Declared

